# Outcomes of aortic aneurysm surgery in England: a nationwide cohort study using hospital admissions data from 2002 to 2015

**DOI:** 10.1186/s12913-019-4755-0

**Published:** 2019-12-23

**Authors:** Ahmed Aber, Thaison Tong, Jim Chilcott, Ravi Maheswaran, Steven M. Thomas, Shah Nawaz, Jonathan Michaels

**Affiliations:** 1 School of Health and Related Research, University of Sheffield, Sheffield, UK; 2 Sheffield Vascular Institute, Sheffied Teaching Hospitals, Sheffield, UK

**Keywords:** Aortic aneurysm, Administrative dataset, Outcomes, Hospital episode statistics

## Abstract

**Background:**

The United Kingdom aortic aneurysms (AA) services have undergone reconfiguration to improve outcomes. The National Health Service collects data on all hospital admissions in England. The complex administrative datasets generated have the potential to be used to monitor activity and outcomes, however, there are challenges in using these data as they are primarily collected for administrative purposes. The aim of this study was to develop standardised algorithms with the support of a clinical consensus group to identify all AA activity, classify the AA management into clinically meaningful case mix groups and define outcome measures that could be used to compare outcomes among AA service providers.

**Methods:**

In-patient data about aortic aneurysm (AA) admissions from the 2002/03 to 2014/15 were acquired. A stepwise approach, with input from a clinical consensus group, was used to identify relevant cases. The data is primarily coded into episodes, these were amalgamated to identify admissions; admissions were linked to understand patient pathways and index admissions. Cases were then divided into case-mix groups based upon examination of individually sampled and aggregate data. Consistent measures of outcome were developed, including length of stay, complications within the index admission, post-operative mortality and re-admission.

**Results:**

Several issues were identified in the dataset including potential conflict in identifying emergency and elective cases and potential confusion if an inappropriate admission definition is used. Ninety six thousand seven hundred thirty-five patients were identified using the algorithms developed in this study to extract AA cases from Hospital episode statistics. From 2002 to 2015, 83,968 patients (87% of all cases identified) underwent repair for AA and 12,767 patients (13% of all cases identified) died in hospital without any AA repair. Six thousand three hundred twenty-nine patients (7.5%) had repair for complex AA and 77,639 (92.5%) had repair for infra-renal AA.

**Conclusion:**

The proposed methods define homogeneous clinical groups and outcomes by combining administrative codes in the data. These methodologically robust methods can help examine outcomes associated with previous and current service provisions and aid future reconfiguration of aortic aneurysm surgery services.

## Background

The United Kingdom had the highest mortality rate for the elective repair of aortic aneurysms (AA) compared to other western European countries in 2007 (7.9% UK vs 3.5% Europe) [1]. Improvement of outcomes such as post-operative mortality following AA repair was a major drive for vascular services reconfiguration in the National Health Service (NHS). Therefore, reliable and consistent methods to obtain comparable data on activity and outcomes could help the NHS measure the success and shortcomings of vascular services reconfigurations. A valuable resource to measure outcomes is the administrative data collected by NHS hospitals [2–4] and there is growing evidence that the quality of this administrative dataset has improved [5–8]. Furthermore, several studies reported that maximum use of the available information in the dataset can improve the validity of outcomes measured [7, 9–11].

NHS England generates Hospital Episode Statistics (HES) that include details of all inpatient admissions, outpatient appointments and accident & emergency attendances at all English NHS hospitals [12]. The HES database provides a detailed source of information regarding patient care across England [5–8]. HES data can be linked to the Office for National Statistics (ONS) mortality registry data and this can help to analyse survival post inpatient discharge. The basic unit of activity measured in HES is the finished consultant episode (FCE). This is a single period of care under one consultant and does not necessarily equate to a single hospital admission, which may comprise more than one episode if care is transferred between consultants or providers. Each FCE contains a primary diagnosis, up to 19 secondary diagnoses and 24 procedure fields. FCEs also include information such as patient demographics, type of admission, source of admission as well as length of stay in critical care and other important clinical and administrative information.

FCEs can be combined to generate a provider spell; this is the period of care when the patient remains in one hospital. This definition does not capture transfers between hospitals during the same stay. Therefore, to generate an accurate admission level dataset for a patient the FCEs need to be combined into a continuous inpatient stay (CIPS). A CIPS starts from the moment a patient is admitted under the care of a consultant in an NHS hospital and includes all the episodes during that admission including transfers to other hospitals [13].

The aim of this study was to develop standardised algorithms with the support of a clinical consensus group to identify all AA activity; classify the AA management into clinically meaningful case mix groups and define outcome measures that could be used to compare outcomes among AA service providers.

## Methods

Vascular related inpatient HES data from the financial year 2002/2003 to 2014/2015 were acquired from NHS Digital data warehouse using broad filters including health resource groups (HRG) codes and office of population census and surveys (OPCS) codes; the OPCS codes are codes for interventions and procedures. The R© programme (Version 3.4.1) (R foundation, Vienna, Austria) was used to develop code to clean, validate, and explore this HES data extract.

From this broad extract of vascular episodes, specific filters were developed based on a combination of the OPCS codes, ICD10 diagnosis codes, and speciality codes to identify all AA-related episodes. The patient identification number (encrypted HESID) for the patients from this extract was used to identify all others episodes for each patient (Appendix 1). These patient identification numbers were also used to link patients to the mortality data from the Office of National Statistics.

The data had already been cleaned and validated to a certain degree at the data warehouse (NHS Digital) before it was passed to us [14]. Nevertheless, significant amount of data cleaning and validating was undertaken prior to analysis (Details of the cleaning and validating steps performed are reported in the Appendix 1 section 2). HES data are well-known for problems with missing data, duplicates, data formatting errors and invalid data [7, 9]**,** but accuracy can be improved by appropriate cleaning and validation.

**Fig. 1:**
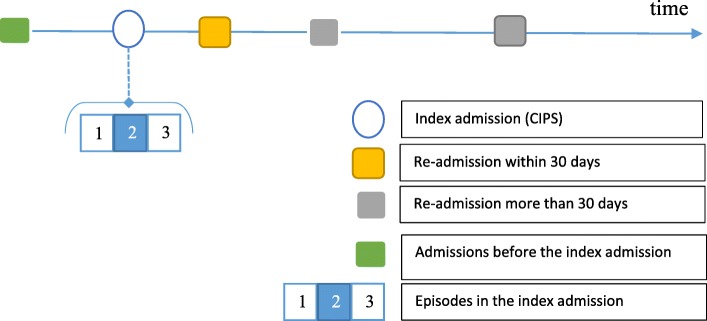
Simplified patient pathway in HES

Patients’ pathways were described in terms of a series of admissions (CIPS) that include single or several episodes for each patient. The index admission was defined as the admission where patients’ received their first AA repair or they died during the index admission secondary to AA without any repair in the current or prior admissions. Figure 1 illustrates a simplified single patient pathway in the data.

To capture all the AA cases and categorise them into clinically meaningful case mix groups an iterative process was employed with input from multidisciplinary group of vascular specialists and data analysts. The clinicians were presented with aggregate data as well as samples of fully anonymised individual records and, based on their recommendations, the algorithms used to define categories were modified. The clinical consensus group divided the AA groups into ‘infra-renal repair’, ‘complex repair’ and ‘AA related death without repair’. The first two groups were subdivided into elective, emergency non-ruptured and ruptured repair subgroups and these were further subdivided into open surgical and endovascular repair (EVAR).

The initial algorithms for identifying the AA repair groups were based upon AA procedure codes (OPCS), whereas for the AA related death with no repair, the algorithm relied on diagnoses codes (ICD-10) (for details of the included codes refer to Tables 11-17 in Appendix 2. The subsequent alterations to the algorithms were based on input from the clinical consensus group and in these alterations, other information from HES and specific codes such as treatment speciality codes [15] (Table 18 in Appendix 2), admission method [16]**,** discharge method [17] were used. These changes to the algorithms were required to overcome coding inconsistencies within AA HES dataset. Key issues identified by the consensus group were:

- Categorisation of cases with multiple, potentially conflicting, OPCS codes.
- Categorisation of admissions into elective and emergency in light of inconsistencies observed when cross tabulating OPCS and ICD-10 codes against admission method.
- Identification of complex AA repaired by vascular specialists.
- Identification of ruptured AA patients who died without AA procedure.
- Distinguishing between of aortic bypass procedures for AA and peripheral arterial disease.
- Identification of index admissions to describe the patient pathway accurately and detect related prior admissions, readmissions and complications.

Following the identification of all the cases within each of the AA case mix groups, outcomes such as length of stay, complications within the index admission, post-operative mortality and re-admission within 30 days of the index admission were calculated for each case mix group.

## Results

**Fig. 2:**
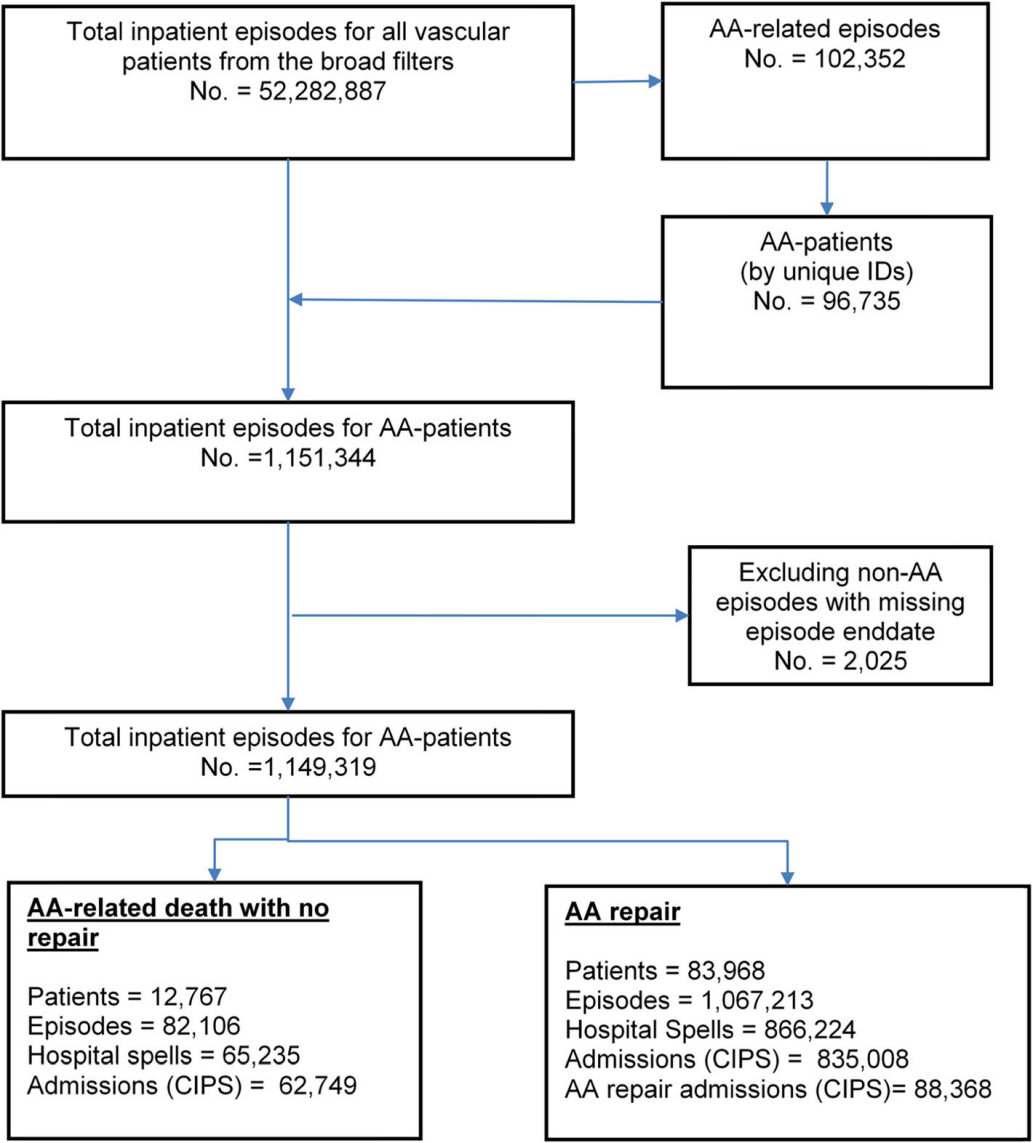
Flow chart for the extraction of AA episodes

The total number of inpatient episodes for vascular patients identified by the broad filters (Appendix 1) between the financial years 2002/2003 and 2014/2015 was 52,282,887. Results of the extraction process to identify AA patients and their episodes and admissions are presented in Fig. 2.

### Developing case mix groups

The development of the case-mix groups was based on the anatomy of the AA disease (infra-renal, complex), admission method (e.g. elective vs emergency), ruptured vs intact AA, type of the procedure (e.g. open repair vs EVAR), and a subgroup of patients dying in-hospital from AA with no previous AA operation. The anatomy of the AA disease was identified by specific OPCS codes that differentiate complex AA procedures from infra-renal AA procedures (See Tables 11-15 in Appendix 2). There were further issues that had to be resolved in the development of case-mix groups.

#### Categorisation of elective and emergency

There were several potential methods to distinguish elective or emergency AA cases. Admission method (admimeth) in HES data defines elective and emergency admissions, OPCS codes differentiate between elective and emergency procedures (See Tables 11-15 in Appendix 2) and ICD-10 codes can describe whether AA is intact or ruptured (See Tables 16-17 in Appendix 2). Table 1 presents cross-tabulation of these three methods for the index AA operation episodes. The table demonstrate the degree of overlap between these methods in identifying elective and emergency episodes in the HES dataset.

**Table 1:** Cross tabulation of different rules to categorise elective and emergency in index episodes

	Admission Method in episodes	
	Emergency admission	Elective Admission
No emergency operations or diagnosis codes	10,082 (36.0%)	54,379 (97.2%)
Emergency ICD-10 codes only (Diagnosis code)	16,384 (58.5%)	1052 (1.9%)
Emergency OPCS codes only (Procedure code)	574 (2.1%)	451 (0.8%)
Both ICD and OPCS emergency codes present	958 (3.4%)	88 (0.2%)
Total	27,998 (100%)	55,970 (100%)

(*Including all index episodes of those with AA repairs: 83968 episodes)

Among cases with emergency admission indicated by the admission method, 36% of them did not have emergency operation or diagnosis codes and 58.5% had only emergency ICD10 codes. Among cases with elective admission indicated by the admission method, 97.2% of them did not have any emergency operation or diagnosis codes. Following discussion with the clinical consensus group based upon examination of sampled cases and aggregate data, admissions were divided into elective and emergency admissions based on admission method, irrespective of the categorisation of the procedure. Further analysis was carried out to consider the identification of ruptured AA based upon ICD10 codes.

#### Categorisation of ruptured aortic aneurysm

There were 27,359 ruptured AA cases identified using the ICD10 diagnosis codes for ruptured AA; of these 1092 were ruptured complex AA repair cases (17.3% of all complex repair cases), 15,717 were ruptured infra-renal repair cases (20.2% of all infra-renal repair cases), and 10,750 were cases of rupture with no definitive repair operation (84.2% of all cases with AA related death with no definitive repair).

For ruptured cases with AA repair (16,809 cases) the in-hospital mortality based on their admission method and the delay (in days) from admission to procedure was investigated and compared to the in-hospital mortality of the non-ruptured AA repair cases. The results are shown in Table 2 and Table 3.

**Table 2:** Cases with ruptured AA diagnostic code (ICD-10) divided by admission method and delay between admission date and operation date

Admission methods	Delay from admission to procedure (days)	Number of cases	In-hospital mortality
Emergency	0	13,392	42.5%
	1	1659	37.9%
	2	290	39.0%
	> = 3 days	809	36.0%
	Missing op dates	26	15.4%
	Total emergency	16,176	42%
Elective	0	201	20.4%
	1	301	25.2%
	2	48	27.1%
	> = 3 days	83	33.7%
	Total elective	633	25.0%
TOTAL cases		16,809	40.9%

**Table 3:** Cases without ruptured ICD-10 codes divided by admission method and delay between admission date and operation date

Admission methods	Delay from admission to procedure (days)	Number of cases	In-hospital mortality
Emergency	0	3733	20%
	1	2358	11%
	2	1015	11%
	> = 3 days	4682	11%
Emergency	Missing op dates	34	18%
	Total emergency	11,822	14%
Elective	0	15,700	3%
	1	33,271	5%
	2	3158	8%
	> = 3 days	3138	9%
Elective	Missing op dates	70	1%
	Total elective	55,337	5%
TOTAL cases		67,159	6%

The mortality rates were significantly higher in those repair cases with the ruptured ICD-10 codes compared with those without these codes. Based on these results, the consensus group concluded that it is appropriate to include all cases that underwent repair and had a ruptured AA ICD-10 codes into a separate case-mix group (ruptured AA) regardless of admission method and delay between admission and operation, with the remaining emergency admissions being treated as a group of “emergency repair without mention of rupture”.

#### Categorisation of open and endovascular AA

The specific procedure codes (OPCS) for endovascular aneurysm repair (EVAR) were introduced in 2005/2006 (See Table 12 in Appendix 2). Prior to this year it is not possible to reliably separate EVAR from open repair. To separate EVAR cases that might have been counted as open repair cases prior to 2005/2006, an approximate approach previously described was used [18]. The algorithm classified AA cases prior to 2005/2006 as EVAR if in addition to the procedure code for open AA repair the episode had codes for insertion of prosthesis into organ and/or aorta specific organ codes or arteriotomy codes (See Table 20 in Appendix 3) [18]**).** Upon investigating these methods, it was found that only the presence of the code for “insertion of prosthesis into organ” was useful in identifying EVAR cases prior to 2005/2006 (For more information see Table 21 in Appendix 3).

Aortic bypass OPCS procedures codes (Table 15 in Appendix 2) could be used to describe AA open repair, however they are also used to describe procedures for occlusive peripheral arterial disease. To separate cases undergoing bypass procedures for occlusive disease from cases of bypass for AA an additional filter was used. Cases with bypass procedures with AA ICD-10 diagnosis codes were added to the open infra-renal AA repair case-mix group. Cases with bypass procedures without such ICD-10 codes were categorised as aortic bypass for peripheral arterial disease.

#### Identifying complex AA procedures performed by vascular specialists

Complex AA open repair procedures could be performed by other specialists including cardiac surgeons, and the same procedure codes (See Tables 13 and 14 in Appendix 2) are used to document these procedures in HES. The cases performed by cardiac/cardio-thoracic surgeons were excluded by adding an extra filter for speciality fields in HES for cardio-thoracic surgery. This filter was applied when identifying AA-related episodes (see Fig. 1).

#### Identifying AA-related deaths without any repair

AA-related deaths without any repair were defined as cases where patients were admitted with a diagnosis of AA and died within the admission, but there was no record of AA repair in that admission or in previous admissions. These cases were identified by including all admissions (CIPS) with a diagnosis of AA and discharge method indicating the patient died in the hospital with no record of AA repair within the admission. Based upon record linkage, those with AA repair within the same or previous admission were excluded.

In total 12,767 cases of AA-related death with no definitive repair were identified from the data between 2002/03 and 2014/15. Table 4 shows the length of time (in days) from the date of admission to the date of death for these patients.

**Table 4:** AA-related deaths without any repair by delay from admission day to death

Admission Method	Delay from admission to death	Number of cases	%
Emergency	0 day	4475	35.1%
	1 day	3246	25.4%
	2 days	1223	9.6%
	> = 3 days	3599	28.2%
Elective	0 day	18	0.1%
	1 day	29	0.2%
	2 days	18	0.1%
	> = 3 days	159	1.2%
TOTAL		12,767	100%

A further investigation was carried to examine the presence of early interventions (not AA repair) in this group. Procedure fields of all episodes within the index admissions were investigated for evidence of early interventions (For more information about early intervention procedures see Table 19 in Appendix 2). The results revealed that only 6.9% of cases had evidence of early interventions (See Table 5).

**Table 5:** AA related death divided based on presence of OPCS codes indicative of early management of ruptured AA

Early interventions OPCS codes	Total number of cases
No early interventions	11,881 (93.1%)
Early interventions	886 (6.9%)
Total	12,767 (100%)

### Comparison of counts with NVR

The UK National Vascular Registry (NVR) is a dataset of vascular procedures performed by vascular surgeons; the information is uploaded voluntarily by vascular specialists and include details of the procedure, information specific to the patient as well as the disease [19]. The annual cases of infra-renal AA elective repairs from HES as identified by the methods described in this paper were compared to the numbers reported by NVR. This group was chosen since it was the only case-mix group consistently reported from 2009 to 2013 [19, 20]. To allow comparability with the NVR cases, all AA operations (not just index admissions) for each patient within the calendar, rather than financial year, were identified. Table 6 shows the comparison between the numbers of elective infra-renal repair cases from HES data and the numbers reported by NVR.

**Table 6:** Comparison of elective infra-renal AA repairs from HES and NVR

Year	2009	2010	2011	2012	2013	Total
NVR cases	4332	4283	4451	4428	4121	21,615
HES cases (Based on the algorithms described in this study)	4666	4441	4743	4631	4603	23,084
% NVR/HES	93%	96%	94%	96%	90%	94%

*NVR collect data in England, Wales, Scotland and Northern Ireland, HES data only cover English hospitals that constitute the majority of the activity. Therefore the missing data from NVR are likely to be higher

### Summary of the identified AA case-mix groups

Ninety six thousand seven hundred and thirty-five patients were identified using the AA case-mix groups described above. From April 2002 to February 2015, 83,968 patients (87% of all cases identified) underwent repair for AA and 12,767 patients (13% of all cases identified) died in hospital without any record of AA repair. Among repair cases, 6329 (7.5%) were repairs for complex AA and 77,639 (92.5%) were infra-renal AA repairs. Within infra-renal AA cases: 15,717 (20.2%) patients had repair for ruptured AA, 51,646 (66.5%) had elective AA repair**,** and 10,276 (13.2%) had emergency or urgent AA repair for non-ruptured aneurysm. For 6329 patients who had repair of complex AA by vascular specialists: 1092 (17.3%) had repair for ruptured AA, 3691 (58.3%) had elective repairs, and 1546 (24.4%) had emergency repairs of non-ruptured AA. Table 7 shows the number of cases by year between 2002/03 and 2014/15.

**Table 7:** Number of open repair and EVAR cases identified by case-mix groups

HES YEAR	INFRA-RENAL AA CASES				Complex AA CASES			
	EVAR		OPEN		EVAR		OPEN	
	cases	%elective	cases	%elective	cases	% elective	cases	% elective
2002/03	58	74%	5517	60%	5	80%	236	52%
2003/04	63	78%	5506	61%	3	67%	307	53%
2004/05	145	77%	5488	61%	16	69%	273	58%
2005/06	260	77%	5459	62%	15	60%	286	59%
2006/07	991	86%	4957	62%	173	64%	301	58%
2007/08	1743	86%	4492	61%	229	65%	233	57%
2008/09	2415	85%	4074	59%	308	62%	218	56%
2009/10	2706	86%	3600	58%	400	67%	187	58%
2010/11	3058	84%	3075	56%	363	66%	193	57%
2011/12	3339	86%	3075	55%	366	61%	204	53%
2012/13	3334	83%	2795	53%	409	63%	224	49%
2013/14	3520	83%	2640	57%	422	62%	213	54%
2014/15	3153	82%	2176	56%	474	62%	271	56%
ALL YEARS	24,785	84%	52,854	59%	3183	64%	3146	56%

HES year starts from beginning of April and end at the last day of March. In the data presented the data for 2014/15 are incomplete and data covers from the start of April to the end of February

### Outcomes in AA case-mix groups

#### Emergency AA repair for intact & ruptured AA and AA-related deaths without definitive repair

**Fig. 3:**
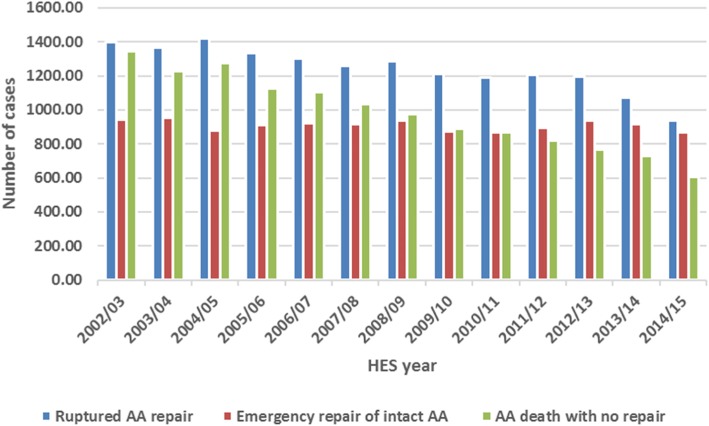
Trend of ruptured AA repair, emergency repair of intact AA and AA-related death with no intervention between April 2002 and February 2015

Figure 3 reports the trend of AA-related death without AA intervention as well as the trends of emergency repair of intact AA and ruptured AA between April 2002 and February 2015.

#### Post-operative mortality

Post-operative mortality may be identified based upon in-hospital mortality, as defined by discharge method [17], or 30-day mortality based upon linked ONS data. The NHS definition of Continuous Inpatient Stay (CIPS) was used to define admission. This captures transfers between hospitals within the same stay. Thus, using this definition, a hospital death is defined as any death within the whole stay. This overcomes the issue where patients die after being transferred from the care of vascular specialist. The CIPS definition still categorises this as an in-hospital death whereas, the NHS definition of hospital spell or episode does not. Table 8 shows the results of mortality outcomes for patients with AA repairs between April 2002 and February 2015. In total 11,111 patients died in hospital following AA repair (13.2% of all AA repairs); however, 30- day mortality following AA was recorded among 10,096 patients (12% of all AA repair cases). The discrepancy between in-hospital mortality and 30-day mortality figures is due to additional mortality amongst those patients remaining in hospitals for longer than 30 days’ post AA repair. Furthermore, using episode to define admission instead of CIPS can lead to significant under-estimation of in-hospital mortality following AA repair.

**Table 8:** Aortic Aneurysm repair mortality outcomes in England between Apr 2002 and Feb 2015

Type of aneurysm	Type of operation	Sub-group	No. Cases	In-hospital mortality based on episodes	In-hospital mortality based on continuous inpatient stay	30-day Mortality
Complex Cases	EVAR	Ruptured	368	25.5%	33.7%	31.0%
		Non-ruptured emergency	836	9.0%	13.2%	11.4%
		Elective	1979	3.6%	5.4%	4.5%
	OPEN REPAIR	Ruptured	724	40.1%	48.3%	44.2%
		Non-ruptured emergency	710	14.4%	19.7%	16.3%
		Elective	1712	9.2%	12.0%	9.8%
Infra-renal Cases	EVAR	Ruptured	1543	18.4%	24.7%	22.8%
		Non-ruptured emergency	2477	3.4%	6.1%	5.2%
		Elective	20,765	1.0%	1.5%	1.7%
	OPEN REPAIR	Ruptured	14,174	34.5%	42.5%	39.4%
		Non-ruptured emergency	7799	11.3%	15.9%	14.0%
		Elective	30,881	4.5%	6.3%	5.5%
TOTAL			83,968	13.2%	12.0%	10.2%

#### Re-admission within 30 days from discharge

Following the index AA repair admission 72,857 patients (86.8% of all AA repair cases) were discharged alive and 10,500 patients (14.41% of all patients discharged alive) had at least one re-admission within 30 days. There were 13,688 30-days readmissions, 9062 patients only had a single 30-day re-admission, and the remaining had more one re-admission within 30 days. Reasons for readmission were summarised by the ICD10 diagnostic blocks. Table 9 shows the main diagnoses for 30-day re-admissions for emergency and elective cases.

**Table 9:** ICD10 blocks for emergency episodes of the 30-day readmissions

ICD10 blocks	Description	Cases	Elective	Emergency	% cases of total
T80-T88	Complications of surgical and medical care, not elsewhere classified	1508	115	1393	14%
R10-R19	Symptoms and signs involving the digestive system and abdomen	747	21	726	7%
I70-I79	Diseases of arteries, arterioles and capillaries	670	247	423	6%
R01-R09	Symptoms and signs involving the circulatory and respiratory systems	537	8	529	5%
K53-K63	Other diseases of intestine	508	35	473	5%
C00-C97	Malignant neoplasms	469	376	93	4%
R50-R69	General symptoms and signs	398	34	364	4%
N30-N39	Other diseases of urinary system	331	26	305	3%
I40-I50	Other forms of heart disease	313	9	304	3%
N17-N19	Renal failure	292	164	128	3%
R30-R39	Symptoms and signs involving the urinary system	285	108	177	3%
J10-J19	Influenza and pneumonia	267	3	264	3%
Z40-Z49	Prophylactic surgery/Adjustment and management of implanted device/ other follow up care… etc.	248	222	26	2%
I20-I25	Ischaemic heart diseases	217	30	187	2%
K90-K93	Other diseases of the digestive system	187	11	176	2%
M50-M54	Other dorsopathies	185	12	173	2%
Z00-Z13	Persons encountering health services for examination and investigation	157	149	8	1%
A00-A09	Intestinal infectious diseases	155	3	152	1%
K50-K52	Non-infective enteritis and colitis	150	5	145	1%
K20-K29	Diseases of oesophagus, stomach and duodenum	141	34	107	1%
I26-I28	Pulmonary heart disease and diseases of pulmonary circulation	132	3	129	1%
I80-I89	Diseases of veins, lymphatic vessels and lymph nodes, not elsewhere classified	131	5	126	1%
J20-J22	Other acute lower respiratory infections	131	1	130	1%
D60-D64	Aplastic and other anaemias	113	28	85	1%
I60-I69	Cerebrovascular disease	103	8	95	1%
A40-A49	Other bacterial diseases	90	5	85	1%
J90-J94	Other diseases of pleura	80	4	76	1%
I10-I15	Hypertensive diseases	48	26	22	0%
N10-N16	Renal tubule-interstitial diseases	41	17	24	0%
D40-D49	Neoplasms of uncertain or unknown behaviour	28	25	3	0%
J95-J99	Other diseases of the respiratory system	23	8	15	0%
D65–69	Coagulation defects, purpura and other haemorrhagic conditions	11	7	4	0%
I51	Complications and ill-defined descriptions of heart disease	5	2	3	0%
H15-H22	Disorders of sclera, cornea, iris and ciliary body	1	1	0	0%
Others	…	1798	300	1498	17%
TOTAL		10,500	2052	8448	100%

Since most of the elective and emergency episodes were relevant to the index admission, the consensus group decided to include all of them in the 30-day readmission analysis.

#### Length of stay and other AA-procedure related complications

Length of hospital stay for an admission is defined as the period (in days) between the starting date of the admission and the discharge date. The short-term post-operative mortality (in-hospital mortality or 30-day mortality) and length of stay already captures certain aspects of the procedure-related complications. There are other methods to identify AA-procedure related complications in the index admissions including the presence of re-operation in the index admission (in addition to mortality and length of stay). Table 10 presents length of stay and reoperation in the index admission (together with the rates of readmissions within 30 days).

**Table 10:** Length of stay, 30-day readmission, and re-op summary for case-mix groups

Type of AA Repair	Type of operation	Sub-group	No. Cases	Index-LOS (median, mean, IQR)	%Read30 (All)	Index re-op
Complex AA Repair Cases	EVAR	Ruptured	368	13; 25.68; (6-31)	16.8%	12.8%
		Non-ruptured emergency	836	19; 34.93; (10-36)	20.2%	11.8%
		Elective	1979	6 ; 11.83 ; (3-10)	19.3%	11.1%
	OPEN REPAIR	Ruptured	724	13; 23.24; (3-28)	8.1%	9.3%
		Non-ruptured emergency	710	21; 34.21; (12-40)	16.2%	19.7%
		Elective	1712	12; 21.1; (8-20)	15.7%	19.6%
Infra-renal AA repair Cases	EVAR	Ruptured	1543	10; 18.6; (5-21)	16.8%	6.9%
		Non-ruptured emergency	2477	10; 18.31;(6-19)	22.1%	4.9%
		Elective	20765	4; 6.6; (3-6)	15.4%	3.0%
	OPEN REPAIR	Ruptured	14174	12; 21.89; (4-26)	8.0%	0.9%
		Non-ruptured emergency	7799	15; 25.1; (10-27)	13.5%	1.3%
		Elective	30881	10; 14.98; (7-15)	10.5%	0.5%
TOTAL			83968	9; 15.71;(5-16)	12.5%	2.6%

## Discussion

This study developed standardised methods to identify AA activity and outcomes in England, based upon the HES administrative dataset. Using a stepwise approach with input from clinicians and data analysts, the solutions aimed to provide consistent and comparable methods to overcome some of the inherent pitfalls and ambiguities in identifying the activity and outcomes associated with AA case mix groups.

Identified issues included invalid, missing, and duplicate information, the limitations of AA diagnostic and procedural codes in separating elective and emergency admissions consistently and the handling of potentially conflicting or overlapping codes [21–34]. Codes for admission methods were more consistent than clinical codes in differentiating between emergency and elective admissions. Mortality rates suggested that previously described methods of combining admission method and delay between admission and procedure [28, 33] were less efficient at identifying ruptured AA repair cases than using the diagnosis codes alone.

Further challenges that were addressed were; changes in coding practices for EVAR prior to the introduction of codes specific to this procedure [28], identification of aortic bypass cases performed for AA as opposed to PAD, identification of complex AA repair treated by vascular specialists, and the identification of cases of AA-related death that had no definitive intervention. The study also demonstrated that potentially valuable co-morbidity data and outcomes measures can be obtained by record linkage, allowing re-operations within the same admission, transfers between specialities, prior and subsequent admissions and long-term mortality. A significant finding from this study is that the choice of episodes or hospital spell to describe index admission for AA repair [21–34] may lead to under-estimation of in-hospital mortality.

An important outcome of this study is the potential standardisation of definitions and algorithms for identifying AA activity and outcomes. Differing definitions used in the past may have resulted in ambiguity, double-counting, misclassification or exclusion of some cases from appropriate case-mix groups, which may have affected the comparability of previous work that used the HES dataset [26, 27, 30–35].

Currently the healthcare quality improvement partnership (HQIP) collects data about AA activity and outcomes through the National Vascular Registry (NVR). NVR dataset contains important information about case complexity as well as risk adjustment, which is not easily available from HES. However, NVR also has drawbacks as it is a voluntary, procedure-based registry, it does not provide information on cases of AA related death with no intervention and contains no data relating to readmissions, repeat procedures or post-discharge outcomes [36]. Although case ascertainment in NVR is high, even small amounts of selectively missing data may distort outcomes. Ideally, linkage between NVR and HES data could address many of the limitations of both datasets. Using the methods described in this paper will help identify AA activity (repair and no definitive intervention) and compare outcomes between different providers and across time.

## Conclusions

HES is a rich source of data but has pitfalls and distortions as shown in this study. These can be overcome by developing consistent methods that rely on the data available within HES to identify and group all relevant AA activity. Many short and long-term outcomes can be analysed by linking admission level data to identify prior and subsequent admissions and by linkage to ONS data. Despite the potential of HES in examining AA activity if inconsistent methods are used, the results can be distorted. HES remains an underused resource for quality assessment of AA services and the use of proposed methods can help identify aortic aneurysm surgery activity and outcomes with greater precision.

## Data Availability

The data that support the findings of this study are available from [NHS Digital] but restrictions apply to the availability of these data, which were used under license for the current study, and so are not publicly available. Data are however available from the authors upon reasonable request and with permission of NHS Digital.

## Appendix 1

### Section One

#### Extract 1

Inpatients

Where HRG 3.5 between Q01and Q99 or between A20 and A23.

Or Any 3 Character Diagnosis in I63, I65, I70, I71, I72, I73, I74, I79, I80, I83, I86, I87.

Or Any 4 Character Diagnosis in D44.6, D75.4, G54.0.

Or Tretspef = 107.

Or Any Opertn between L16 and L99 or between X07 and X12.

Or Any 3 Character Opertn in W06, U11.

Or Any 4 Character Opertn in Z36.1, Z40.3.

Outpatients

Where Any Opertn between L16 and L99.

Or Tretspef = 107.

#### Extract 2

Filter on HESIDS produced in Extract 1 for Inpatients and Outpatients across both datasets.

### Section Two

Cleaning steps:

- Standard HES cleaning:
  - Problems with dates
    - Date Format
    - Missing Dates (admidate, epistart, and epiend)
    - Invalid Dates
  - Problems with diagnosis fields and operation fields
- Concurrent episodes
- Inconsistent Date of Birth
- Patient died more than once (error with dismeth code) (15 cases – one of them died four times)

## Appendix 2

**Table 11:** OPCS codes for open repair of infra-renal AA

OPCS Code	OPCS Description
L184	Emergency replacement of aneurysmal segment of infrarenal abdominal aorta by anastomosis of aorta to aorta
L185	Emergency replacement of aneurysmal segment of abdominal aorta by anastomosis of aorta to aorta NEC
L186	Emergency replacement of aneurysmal bifurcation of aorta by anastomosis of aorta to iliac artery
L188	Other specified emergency replacement of aneurysmal segment of aorta
L189	Unspecified emergency replacement of aneurysmal segment of aorta
L194	Replacement of aneurysmal segment of infrarenal abdominal aorta by anastomosis of aorta to aorta NEC
L195	Replacement of aneurysmal segment of abdominal aorta by anastomosis of aorta to aorta NEC
L196	Replacement of aneurysmal bifurcation of aorta by anastomosis of aorta to iliac artery NEC
L198	Other specified other replacement of aneurysmal segment of aorta
L199	Unspecified other replacement of aneurysmal segment of aorta
L204	Emergency bypass of segment of infrarenal abdominal aorta by anastomosis of aorta to aorta NEC
L205	Emergency bypass of segment of abdominal aorta by anastomosis of aorta to aorta NEC
L214	Bypass of segment of infrarenal abdominal aorta by anastomosis of aorta to aorta NEC
L215	Bypass of segment of abdominal aorta by anastomosis of aorta to aorta NEC
L254	Operations on aneurysm of aorta NEC
L481	Emergency replacement of aneurysmal common iliac artery by anastomosis of aorta to common iliac artery
L482	Emergency replacement of aneurysmal iliac artery by anastomosis of aorta to external iliac artery
L483	Emergency replacement of aneurysmal artery of leg by anastomosis of aorta to common femoral artery
L484	Emergency replacement of aneurysmal artery of leg by anastomosis of aorta to superficial femoral artery
L485	Emergency replacement of aneurysmal iliac artery by anastomosis of iliac artery to iliac artery
L486	Emergency replacement of aneurysmal artery of leg by anastomosis of iliac artery to femoral artery
L488	Other specified emergency replacement of aneurysmal iliac artery
L489	Unspecified emergency replacement of aneurysmal iliac artery
L491	Replacement of aneurysmal common iliac artery by anastomosis of aorta to common iliac artery NEC
L492	Replacement of aneurysmal iliac artery by anastomosis of aorta to external iliac artery NEC
L493	Replacement of aneurysmal artery of leg by anastomosis of aorta to common femoral artery NEC
L494	Replacement of aneurysmal artery of leg by anastomosis of aorta to superficial femoral artery NEC
L495	Replacement of aneurysmal iliac artery by anastomosis of iliac artery to iliac artery NEC
L496	Replacement of aneurysmal artery of leg by anastomosis of iliac artery to femoral artery NEC
L498	Other specified other replacement of aneurysmal iliac artery
L499	Unspecified other replacement of aneurysmal iliac artery
L533	Operations on aneurysm of iliac artery NEC

**Table 12:** OPCS codes for endovascular repair of infra-renal AA

OPCS Code	OPCS Description
L271	Endovascular insertion of stent graft for infrarenal abdominal aortic aneurysm
L275	Endovascular insertion of stent graft for aortic aneurysm of bifurcation NEC
L276	Endovascular insertion of stent graft for aorto-uniiliac aneurysm
L278	Other specified transluminal insertion of stent graft for aneurysmal segment of aorta
L279	Unspecified transluminal insertion of stent graft for aneurysmal segment of aorta
L281	Endovascular insertion of stent for infrarenal abdominal aortic aneurysm
L285	Endovascular insertion of stent for aortic aneurysm of bifurcation NEC
L286	Endovascular insertion of stent for aorto-uniiliac aneurysm
L288	Other specified transluminal operations on aneurysmal segment of aorta
L289	Unspecified transluminal operations on aneurysmal segment of aorta

**Table 13:** OPCS codes for open repair of complex AA

OPCS Code	OPCS Description
L181	Emergency replacement of aneurysmal segment of ascending aorta by anastomosis of aorta to aorta
L182	Emergency replacement of aneurysmal segment of thoracic aorta by anastomosis of aorta to aorta NEC
L183	Emergency replacement of aneurysmal segment of suprarenal abdominal aorta by anastomosis of aorta to aorta
L191	Replacement of aneurysmal segment of ascending aorta by anastomosis of aorta to aorta NEC
L192	Replacement of aneurysmal segment of thoracic aorta by anastomosis of aorta to aorta NEC
L193	Replacement of aneurysmal segment of suprarenal abdominal aorta by anastomosis of aorta to aorta NEC
L201	Emergency bypass of segment of ascending aorta by anastomosis of aorta to aorta NEC
L202	Emergency bypass of segment of thoracic aorta by anastomosis of aorta to aorta NEC
L203	Emergency bypass of segment of suprarenal abdominal aorta by anastomosis of aorta to aorta NEC
L211	Bypass of segment of ascending aorta by anastomosis of aorta to aorta NEC
L212	Bypass of segment of thoracic aorta by anastomosis of aorta to aorta NEC
L213	Bypass of segment of suprarenal abdominal aorta by anastomosis of aorta to aorta NEC
L221	Revision of prosthesis of thoracic aorta

**Table 14:** OPCS codes for endovascular repair of complex AA

OPCS Code	OPCS Description
L266	Transluminal aortic stent graft with fenestration NEC
L267	Transluminal aortic branched stent graft NEC
L272	Endovascular insertion of stent graft for suprarenal aortic aneurysm
L273	Endovascular insertion of stent graft for thoracic aortic aneurysm
L274	Endovascular insertion of stent graft for aortic dissection in any position
L282	Endovascular insertion of stent for suprarenal aortic aneurysm
L283	Endovascular insertion of stent for thoracic aortic aneurysm
L284	Endovascular insertion of stent for aortic dissection in any position

**Table 15:** OPCS codes for Aorto-iliac bypass, aortic/iliac endarterectomy or repair

OPCS Code	OPCS Description
L206	Emergency bypass of bifurcation of aorta by anastomosis of aorta to iliac artery NEC
L208	Other specified other emergency bypass of segment of aorta
L209	Unspecified other emergency bypass of segment of aorta
L216	Bypass of bifurcation of aorta by anastomosis of aorta to iliac artery NEC
L218	Other specified other bypass of segment of aorta
L219	Unspecified other bypass of segment of aorta
L222	Revision of prosthesis of bifurcation of aorta
L223	Revision of prosthesis of abdominal aorta NEC
L224	Removal of prosthesis from aorta
L228	Other specified attention to prosthesis of aorta
L229	Unspecified attention to prosthesis of aorta
L231	Plastic repair of aorta and end to end anastomosis of aorta
L232	Plastic repair of aorta using subclavian flap
L233	Plastic repair of aorta using patch graft
L238	Other specified plastic repair of aorta
L239	Unspecified plastic repair of aorta
L251	Endarterectomy of aorta and patch repair of aorta
L252	Endarterectomy of aorta NEC
L255	Operations on aortic body
L258	Other specified other open operations on aorta
L259	Unspecified other open operations on aorta
L501	Emergency bypass of common iliac artery by anastomosis of aorta to common iliac artery NEC
L502	Emergency bypass of iliac artery by anastomosis of aorta to external iliac artery NEC
L503	Emergency bypass of artery of leg by anastomosis of aorta to common femoral artery NEC
L504	Emergency bypass of artery of leg by anastomosis of aorta to deep femoral artery NEC
L505	Emergency bypass of iliac artery by anastomosis of iliac artery to iliac artery NEC
L506	Emergency bypass of artery of leg by anastomosis of iliac artery to femoral artery NEC
L508	Other specified other emergency bypass of iliac artery
L509	Unspecified other emergency bypass of iliac artery
L511	Bypass of common iliac artery by anastomosis of aorta to common iliac artery NEC
L512	Bypass of iliac artery by anastomosis of aorta to external iliac artery NEC
L513	Bypass of artery of leg by anastomosis of aorta to common femoral artery NEC
L514	Bypass of artery of leg by anastomosis of aorta to deep femoral artery NEC
L515	Bypass of iliac artery by anastomosis of iliac artery to iliac artery NEC
L516	Bypass of artery of leg by anastomosis of iliac artery to femoral artery NEC
L518	Other specified other bypass of iliac artery
L519	Unspecified other bypass of iliac artery
L521	Endarterectomy of iliac artery and patch repair of iliac artery
L522	Endarterectomy of iliac artery NEC
L528	Other specified reconstruction of iliac artery
L529	Unspecified reconstruction of iliac artery
L531	Repair of iliac artery NEC
L538	Other specified other open operations on iliac artery
L539	Unspecified other open operations on iliac artery
L651	Revision of reconstruction involving aorta
L652	Revision of reconstruction involving iliac artery

**Table 16:** ICD-10 codes for aortic aneurysm rupture

ICD-10 Code	ICD-10 Description
I711	Thoracic aortic aneurysm, ruptured
I713	Abdominal aortic aneurysm, ruptured
I715	Thoracoabdominal aortic aneurysm, ruptured
I718	Aortic aneurysm of unspecified site, ruptured

**Table 17:** ICD-10 codes for aortic aneurysm with no mention of rupture

ICD-10 Code	ICD-10 Description
I712	Thoracic aortic aneurysm, without mention of rupture
I714	Abdominal aortic aneurysm, without mention of rupture
I716	Thoracoabdominal aortic aneurysm, without mention of rupture
I719	Aortic aneurysm of unspecified site, without mention of rupture

**Table 18:** Hospital episode statistics treatment speciality codes

Code	Speciality
100	GENERAL SURGERY
103	BREAST SURGERY
104	COLORECTAL SURGERY
105	HEPATOBILIARY & PANCREATIC SURGERY
106	UPPER GASTROINTESTINAL SURGERY
107	VASCULAR SURGERY
170	CARDIOTHORACIC SURGERY
172	CARDIAC SURGERY
173	THORACIC SURGERY

**Table 19:** OPCS codes for possible early interventions

Y502	Laparotomy approach NEC
Y701	Emergency operations NOC
L704	Open cannulation of artery
L911	Open insertion of central venous catheter
L912	Insertion of central venous catheter NEC
L913	Attention to central venous catheter NEC
L914	Removal of central venous catheter
L915	Insertion of tunnelled venous catheter
U011	Computed tomography of whole body
U012	Magnetic resonance imaging of whole body
U018	Other specified diagnostic imaging of whole body
U019	Unspecified diagnostic imaging of whole body
U071	Computed tomography of chest
U072	Magnetic resonance imaging of chest
U078	Other specified diagnostic imaging of chest
U079	Unspecified diagnostic imaging of chest
U081	Computed tomography of abdomen NEC
U082	Ultrasound of abdomen
U085	Magnetic resonance imaging of abdomen
U089	Unspecified diagnostic imaging of abdomen
U091	Computed tomography of pelvis
U113	Vascular ultrasound NEC
U117	Magnetic resonance angiography
U118	Other specified diagnostic imaging of vascular system
U119	Unspecified diagnostic imaging of vascular system
U216	Ultrasound scan NEC
U218	Other specified diagnostic imaging procedures
U219	Unspecified diagnostic imaging procedures

## Appendix 3

**Table 20:** Cases with ruptured AA diagnostic code (ICD-10) divided by admission method and delay between admission date and operation date

Admission methods	Delay from admission to procedure (days)	Number of cases	In-hospital mortality
Emergency	0	13,392	42.5%
	1	1659	37.9%
	2	290	39.0%
	> = 3 days	809	36.0%
	Missing op dates	26	15.4%
	Total emergency	16,176	42%
Elective	0	201	20.4%
	1	301	25.2%
	2	48	27.1%
	> = 3 days	83	33.7%
	Total elective	633	25.0%
TOTAL cases		16,809	40.9%

**Table 21:** Cases without ruptured ICD-10 codes divided by admission method and delay between admission date and operation date

Admission methods	Delay from admission to procedure (days)	Number of cases	In-hospital mortality
Emergency	0	3733	20%
	1	2358	11%
	2	1015	11%
	> = 3 days	4682	11%
Emergency	Missing op date*s*	34	18%
	Total emergency	11,822	14%
Elective	0	15,700	3%
	1	33,271	5%
	2	3158	8%
	> = 3 days	3138	9%
Elective	Missing op dates	70	1%
	Total elective	55,337	5%
TOTAL cases		67,159	6%

**Table 22:** OPCS codes for procedures used in combination with open OPCS codes to identify EVAR cases not coded with Table 14 codes of Appendix 2.

OPCS Code	OPCS Description
Y022	Insertion of prosthesis into organ NOC
Y532/Y782	Arteriotomy approach to organ using image guidance with computed tomography
Y532/Y783	Arteriotomy approach to organ using image guidance with ultrasound
Z341	Ascending aorta
Z342	Aortic arch
Z343	Descending thoracic aorta
Z344	Thoracic aorta NEC
Z345	Suprarenal abdominal aorta
Z346	Infrarenal abdominal aorta
Z347	Abdominal aorta NEC

The use of ‘insertion of prosthesis into organ NOC’ code alongside open AA repair code increased between 2002/03 and 2008/09, however this trend reversed between 2008/09 and 2014/15. Comparing the in-hospital mortality between the AA cases using ‘insertion of prosthesis” along the AA repair codes with open AA cases with open AA repair codes only; there is a clear difference between the two groups. The in-hospital mortality and percentage of elective for OPEN cases with the Y022 code before 2008/09 look like cases with the new EVAR codes. This observation is not present in the years after 2008/09.Based on this observation, the consensus group decided to classify open AA cases with the ‘insertion of prosthesis’ code as EVAR for the years before 2008/09.

**Table 23:** Using the insertion of prosthesis in organ code to identify EVAR from open repairs in early years

	OPEN AA cases without “insertion of prosthesis in organ” code			OPEN AA cases with “insertion of prosthesis in organ” code			Cases with the new EVAR codes		
Year	Number	In-hospital Mortality	Percentage of elective	Number	In-hospital Mortality	Percentage of elective	Number	In-hospital Mortality	Percentage of elective
2002/03	5753	20.84%	60%	63	11.11%	75%			
2003/04	5813	19.34%	60%	66	4.55%	77%			
2004/05	5761	18.57%	61%	161	9.32%	76%			
2005/06	5745	16.99%	62%	275	9.45%	76%			
2006/07	5258	16.83%	62%	160	6.25%	82%	1004	5.18%	83%
2007/08	4725	16.23%	61%	171	7.60%	80%	1801	4.55%	84%
2008/09	4292	17.61%	59%	180	6.67%	73%	2543	4.40%	83%
2009/10	3743	16.54%	58%	44	13.64%	57%	3106	4.54%	84%
2010/11	3247	16.94%	56%	21	19.05%	43%	3421	4.15%	82%
2011/12	3264	16.85%	55%	15	53.33%	33%	3705	3.99%	84%
2012/13	3011	16.57%	53%	8	37.50%	38%	3743	3.50%	81%
2013/14	2846	16.65%	57%	7	42.86%	14%	3942	3.58%	81%
2014/15	2439	17.43%	56%	8	25.00%	38%	3627	4.36%	80%

This includes all repair cases

**Table 24:** AA related death divided based on presence of OPCS codes indicative of early management of ruptured AA

Early interventions OPCS codes	Total number of cases
No early interventions	11,881 (93.1%)
Early interventions	886 (6.9%)
Total	12,767 (100%)

## Abbreviations

AA: Aortic aneurysm
Admimeth: Admission method
CIPS: Continuous inpatient stay
EVAR: Endovascular repair
FCE: Finished consultant episode
HES: Hospital episode statistics
HRG: Health resource groups
ICD10 Codes: International classification of diseases, 10th
NVR: UK National vascular registry
ONS: Office for National Statistics
OPCS: Office of population census and surveys
